# The Multidisciplinary Management of Avulsed Teeth: A Case Report

**Published:** 2012-10-13

**Authors:** Adriana de Jesus Soares, Maíra do Prado, Thiago Farias Rocha Lima, Brenda Paula Figueiredo de Almeida Gomes, Alexandre Augusto Zaia, Francisco José de Souza-Filho

**Affiliations:** 1. Department of Restorative Dentistry, Endodontics Area, State University of Campinas-UNICAMP, Piracicaba, SP, Brazil

**Keywords:** Orofacial Accident, Tooth Avulsion, Tooth Replantation, Tooth Resorption, Tooth Mobility, Traumatic Injury

## Abstract

This paper reports multidisciplinary treatment of a dental trauma case to achieve a favorable prognosis. A healthy 14-year-old girl reported avulsion of teeth 11 and 21 which had occurred three months earlier. The initial treatment consisting of replantation with a semi-rigid splint was performed in hospital. At presentation, the patient was still using the semi-rigid splint. The clinical examination revealed the presence of increased mobility in teeth 11 and 21, and absence of vitality in both. Radiographic examination showed the presence of inflammatory external root resorption in both teeth. The treatment proposed consisted of teeth extraction, a temporary prosthesis followed by adhesive prosthesis, and finally, implant surgery associated with porcelain crowns.

## Introduction

Tooth avulsion is the complete displacement of a tooth from its socket due to accidental or non-accidental injury [1]. It rarely occurs, representing only about 0.5% to 3.0% of all traumatic injuries [2]. The most frequently affected teeth are the upper central incisors in children between the ages of 7 and 9 years [3].

Replantation of the avulsed tooth is considered the best aesthetic and functional solution [4]. However, the decision to replant the teeth depends on several factors: periodontal damage, intact alveolar socket, extra-alveolar period, and stage of root development [5]. After replantation of the avulsed tooth, root resorption becomes the greatest danger to the tooth, occurring in 50% to 76% of cases [6][7][8]. It is associated with length of the extra-alveolar storage period and the storage medium [1][9]. Root resorption mechanisms can be associated with damage to the cementum layer, exposing the root surface to osteoclasts, which in turn can resorb the dentin [2][5][10].

Root resorption can be treated with calcium hydroxide, but it can be prevented by the early removal of necrotic pulp and dressing the root canal with calcium hydroxide followed by gutta-percha/sealer obturation [11]. An alternative approach involves the use of Ledermix paste as the initial intra-canal medicament to act as an anticlastic agent. This root canal medication is replaced at six-weekly intervals for a period of approximately three months; it is also replaced where there is radiographic signs of resorption [9]. MTA has also been used in root resorption treatment [12].

This paper reports a case of dental trauma in which a multidisciplinary collaboration was needed to achieve better planning and a more favorable prognosis.

## Case report

A healthy 14-year-old girl presented herself at the Dental Trauma Service of the Faculty of Dentistry of Piracicaba in March 2006. She reported displacement of the upper central incisors during a bicycle accident three months earlier (December 2005). The first treatment had been performed in a hospital, where the teeth were replanted and splinted with a semi-rigid splint. The patient also reported that the extra-alveolar period lasted about 3 hours and 10 minutes, with the teeth being in dry storage for 10 minutes after being stored in saline solution.

**Figure 1:**
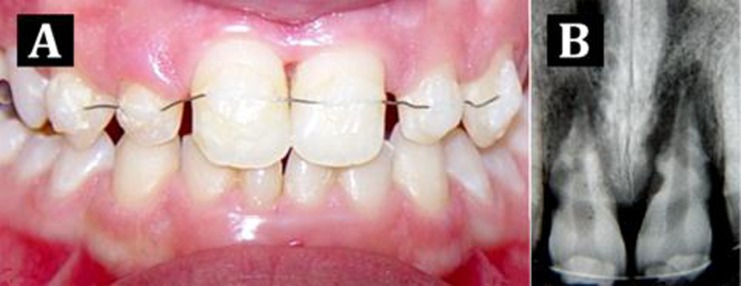
Initial clinical and radiographic aspects

At the clinical examination, the patient was still using the semi-rigid splint (Figure 1A). Percussion, palpation and pulp vitality test (cold test) were done from canine to canine and revealed the presence of increased mobility as well as absence of vitality in teeth 11 and 21. Radiographic examination (Figure 1B) showed the presence of inflammatory external root resorption in both teeth.

First, the teeth were accessed and instrumented using a crown-down technique with 2% chlorhexidine gel as chemical auxiliary substance; saline solution was used for irrigating the canal between files. Once the preparation was finished, during insertion of calcium hydroxide medicament, the patient reported pain and extravasations of the intracanal medication were observed at the gingival sulcus.

A consultation was held with specialists in endodontics, oral surgery, prosthodontics, and periodontics. Based on the clinical conditions (i.e. presence of severe inflammatory root resorption, showing communication with periodontal tissue associated with enhanced tooth mobility), the teeth were extracted and a prosthesis was placed in the region, with implants being indicated in the future. Treatment and this case report were performed with the patient’s consent.

**Figure 2:**
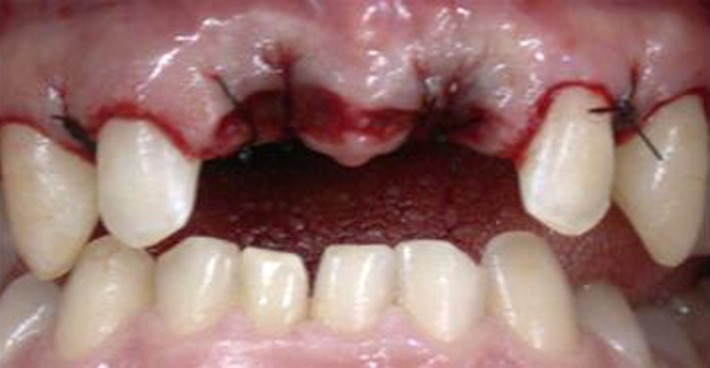
Clinical aspect after extraction

**Figure 3:**
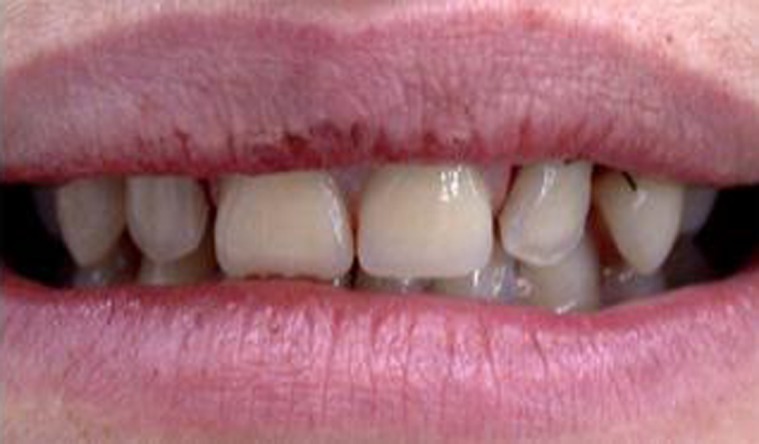
Clinical aspect of the removable prosthodontic appliance

The proposed treatment consisted of dental extraction (Figure 2) and use of a removable prosthodontic appliance (Figure 3).

In the first session, the patient also underwent cosmetic periodontal surgery involving the upper lateral incisors, canines, and first premolars in order to improve her gingival aesthetics.

**Figure 4:**
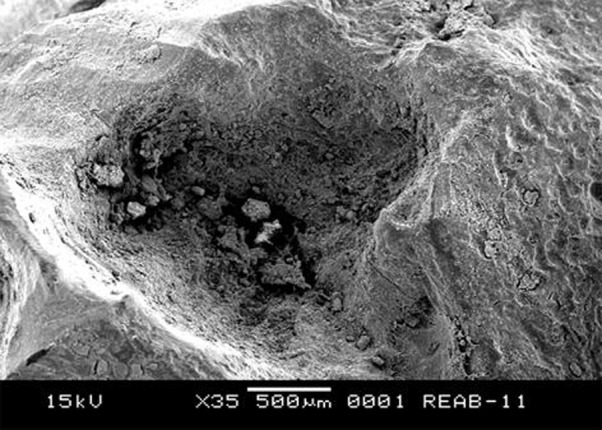
SEM image of root resorption craters

After extraction, the roots were separated from the crowns and evaluated in a scanning electron microscopy where root resorption craters were observed (Figure 4).

**Figure 5:**
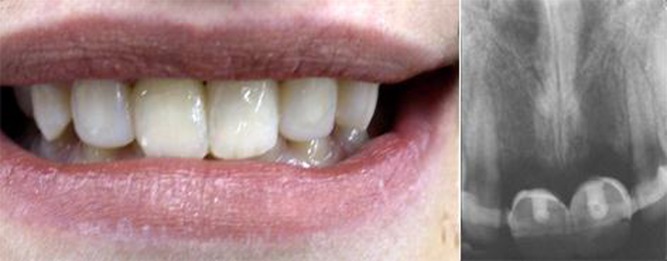
Clinical and radiograph aspects of the adhesive prosthesis

The crowns were cleaned and stored in saline solution. One week later, the sutures were removed. Following 45 days of periodontal healing, an adhesive prosthesis was prepared (Figure 5).

It consisted of a slice on the palatal side of the central incisors in association with a polyethylene fiber (Ribbond Reinforcement. Ribbon, Ribbon Inc., Seattle, WA, USA) to fix the teeth. Next, the lateral incisors and canines were etched with 37% phosphoric acid and hybridized with an adhesive system (Single Bond, 3M/ESPE, St. Paul, MN, USA), with these teeth being fixed to the central incisors with other Ribbond fiber in association with a Resin composite (Z250, 3M ESPE, St Paul, MN, USA). The patient wore this adhesive prosthesis for three years.

**Figure 6:**
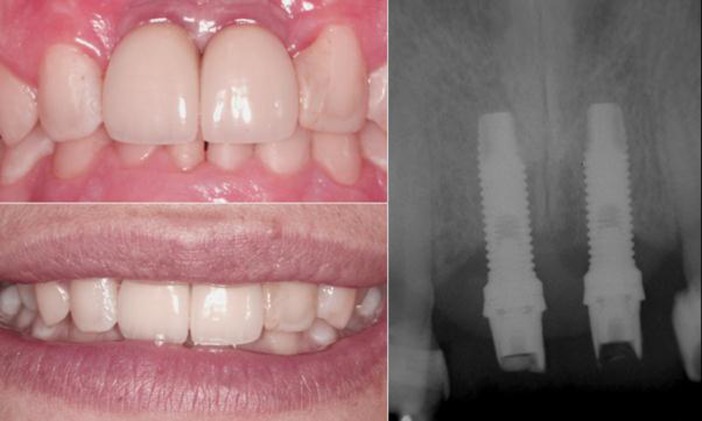
Clinical and radiographic aspect of the final treatment

In 2009, when our patient had turned 17, the patient was referred to an oral surgeon to insert dental implants to replace 11 and 21. After the implant surgery, the patient used temporary crowns for 6 months and then definitive individual porcelain crowns were made. Figure 6 shows the clinical and radiographic aspects of the final treatment.

## Discussion

One of the most challenging problems in dentistry is deciding which treatment option is best, i.e. the decision to replace one or more maxillary incisors that have been lost as a result of traumatic injuries. An optimal outcome frequently involves an interdisciplinary team of experts, including periodontists, endodontists, oral surgeons, orthodontists, and prosthodontists. The treatment is typically complex and the prognosis often uncertain. There are multiple solutions available to treat this kind of problem. The ideal treatment is usually the most conservative option, in which individual aesthetics and functional requirements are met [13]. In this report, both the case study and the treatment were conducted by an endodontist, a periodontist, an oral surgeon, and a prosthodontist.

Treatment plans for inflammatory external root resorption include long-term calcium hydroxide therapy, Ledermix Paste, and MTA obturation [9][11][12]. In the present case, a calcium hydroxide therapy was initially used; however, this treatment was not concluded due to the endo-periodontal communication observed during placement of the intracanal medication. After extraction, it was possible to confirm the endo-periodontal communication. In fact, cone beam computed tomography could be an auxiliary diagnostic tool to detect this communication before extraction [14]. However, this would not have had any influence on the treatment planning proposed for this case.

The cosmetic periodontal surgery could have been performed later, that is, at the moment of implant surgery but in order to optimize the aesthetic immediately the periodontist decided to perform it at the moment of extraction.

The patient’s own natural crown was used as an adhesive prosthesis because it is a low-cost treatment and aesthetically pleasing. Ribbond fiber was used in association with composite resin due to its good properties of elasticity, translucency, adaptability, tenaciousness, and resistance to traction and impact, thus increasing the mechanical strength of the composite, including impact, flexural resistance and modulus elasticity [15]. These qualities are favorable for traumatized anterior teeth. After the adhesive prosthesis cementation, the patient was followed up for three years and made visits annually.

After her growth spurt [5][16][17][18], the patient was referred to an oral surgeon to finalize the treatment with two implants and individual porcelain crowns.

The necessity for an interdisciplinary approach to the treatment of anterior tooth injury has been long emphasized. In addition, it is clear that without cooperation among the disciplines, treatment of such will not be favorable [13][19].

This paper reported a case of avulsion associated with severe inflammatory external resorption, where a multidisciplinary approach was essential to the success of the case. Preventive campaigns, including how and where to store a tooth and its replacement, are essential for improving the prognosis of avulsion cases.
